# Diagnostic Accuracy of Antigen 5-Based ELISAs for Human Cystic Echinococcosis

**DOI:** 10.1371/journal.pntd.0004585

**Published:** 2016-03-29

**Authors:** Daniela Pagnozzi, Maria Filippa Addis, Grazia Biosa, Anna Maria Roggio, Vittorio Tedde, Mara Mariconti, Francesca Tamarozzi, Valeria Meroni, Gabriella Masu, Giovanna Masala, Enrico Brunetti, Sergio Uzzau

**Affiliations:** 1 Porto Conte Ricerche Srl, Tramariglio, Alghero (Sassari), Italy; 2 Department of Clinical, Surgical, Diagnostic and Pediatric Sciences, University of Pavia, Pavia, Italy; 3 WHO Collaborating Centre for the Clinical Management of Cystic Echinococcosis, Pavia, Italy; 4 Department of Microbiology and Virology, IRCCS San Matteo Hospital Foundation and Department of Internal Medicine and Clinical Therapy, University of Pavia, Pavia, Italy; 5 Centro Nazionale di Riferimento per l’Echinococcosi, IZS “G. Pegreffi”, Sassari, Italy; 6 Division of Infectious and Tropical Diseases, IRCCS San Matteo Hospital Foundation, Pavia, Italy; University of Zurich, SWITZERLAND

## Abstract

**Background:**

Clinical diagnosis and follow up of cystic echinococcosis (CE) are based on imaging complemented by serology. Several immunodiagnostic tests are commercially available, but the development of new tools is still needed to overcome the lack of standardization of the target antigen, generally consisting of a crude extract of *Echinococcus granulosus* hydatid cyst fluid. In a previous work, we described a chromatographic method for the preparation of a highly enriched Antigen 5 fraction from hydatid cyst fluid. The high reactivity of patient sera against this preparation prompted us to evaluate further this antigen for the serodiagnosis of CE on a larger cohort of samples.

**Methodology/Principal Findings:**

A total of 327 sera from CE patients with heterogeneous conditions for cyst stage, cyst number, organ localization, drug therapy, and surgical intervention, together with 253 sera from healthy controls, were first analyzed by an ELISA based on the Ag5 preparation in two different experimental setups and, in parallel, by a commercial ELISA routinely used in clinical laboratories for CE serodiagnosis. The Ag5 ELISAs revealed different sensitivity (88.3% vs 95.3%) without significant differences in specificity (94.1% vs 92.5%), for the two setups, respectively. Moreover, possible relationships between the Ag5 ELISA absorbance results and clinical variables were investigated. Chi squared test, bivariate logistic regression and multiple regression analyses highlighted differences in the serology reactivity according to pharmacological treatment, cyst activity, and cyst number.

**Conclusions/Significance:**

The two Ag5 ELISAs revealed different performances depending on the setup. The good diagnostic sensitivity and the high reliability of the Ag5 preparation method make this antigen a promising candidate for the serodiagnosis of CE. Further studies will be needed to evaluate the ability of our test to provide useful information on specific CE clinical traits.

## Author Summary

Cystic echinococcosis is a neglected disease caused by the larval stage of the tapeworm *Echinococcus granulosus* complex affecting both humans and livestock. The disease is considered one of the world’s major zoonoses, and represents a public health problem. Clinical diagnosis and follow-up is mainly based on imaging, while serology should complement imaging-based diagnosis when imaging features are unclear. However, current commercial immunoassays lack satisfactory sensitivity and specificity. They are mostly based on crude antigen preparations of *E*. *granulosus* hydatid cyst fluid, a heterogeneous mixture containing molecules of both parasite and host origin, thus standardization is also an issue. Ag5 is one of the most immunogenic proteins present in the hydatid cyst fluid. In a previous work, we described a method enabling the preparation of a highly enriched Ag5 fraction. Here, we present the evaluation of the diagnostic performances of this preparation in two ELISA setups, using a large number of human sera. The influence of several clinical variables on the performance of the tests was also assessed. The results obtained by the Ag5 ELISAs, combined with the robustness of the Ag5 preparation method, make this antigen a promising candidate for the serodiagnosis of CE.

## Introduction

Cystic echinococcosis (CE) is a neglected zoonotic disease caused by the larval form of the tapeworm *Echinococcus granulosus* complex. The definitive hosts are dogs and other canids, while sheep and other livestock are the natural intermediate hosts; humans are occasional intermediate hosts. Intermediate hosts can be infected by ingestion of food and water contaminated with the parasite eggs eliminated with the feces of infected dogs.

The early phase of infection is generally asymptomatic. Small, well encapsulated, viable cysts or old cysts with pseudosolid content typically do not induce major pathology, and patients may remain asymptomatic for years or even permanently. This is likely the reason why almost 50% of CE patients recorded in the Italian Hospital Discharge Records have been diagnosed accidentally during investigations for other diseases, and 57% of cases are people over 60 years old [1].

CE has many important economic effects, the most evident and tangible of which is the cost of expensive medical treatment for human cases; moreover, there is also a strong negative impact on the economy due to the large diffusion of CE among livestock [2–3]. Currently, CE can be treated according to four different approaches: surgery, percutaneous techniques, chemotherapy with benzimidazoles, and with a “watch and wait” approach for inactive cysts. Unfortunately, 20%–40% of the patients respond only temporarily to chemotherapy, and revert to their previous stage (mainly CE2 and CE3b) after the end of treatment [4].

The most affected regions include the Mediterranean, Eastern Europe, parts of South America, parts of Africa, and Central Asia/Western China. In Italy, the average annual incidence rate of hospital cases (AIh) between 2001 and 2012 was 1.6/10^5^ inhabitants [1].

At present, no marker of cyst viability and therapy efficacy exists, and serology may remain positive for years even after successful therapy. As a consequence, long-term follow-up with imaging is required for the clinical management of patients.

It is therefore important to invest in innovative technologies that facilitate the monitoring and control of this infectious disease in humans and in farm animals.

Currently, CE diagnosis in humans is mostly based on imaging techniques [5], and the clinical approach is based on the WHO international classification of ultrasound images according to the stage of the cyst: CE1, CE2, CE3b (active), CE3a (transitional), and CE4 and CE5 (inactive) [6–8]. Ultrasonography (US), due to the relatively low cost and size of the equipment, is easily transportable in remote resource-poor areas, provides a useful tool for screening, clinical diagnosis, and cyst monitoring [9–11]. However, serology has an important role in supporting the diagnosis of CE, since serological tests are generally cheap, quick, and require less trained and specialized personnel for result interpretation. This is particularly true in areas where US expertise in the diagnosis of CE is scant and/or not easily accessible, and when lesions do not show pathognomonic signs of a parasitic origin, such as young CE1 cysts or inactive CE4–CE5 cysts. Unfortunately, these stages are also those with a broader differential diagnosis (e.g. with simple cysts, neoplastic lesions) whose serology results are also difficult to interpret [12–13].

Commercially available immunoassays are mostly based on hydatid cyst fluid (HCF), collected from infected animals. However, the heterogeneity of this preparation negatively impacts on the sensitivity and specificity of the tests. Many purified native, recombinant, or synthetic antigen preparations have been tested in the last decade, although with controversial results [14–16]. This is most likely due to the poor inter-laboratory reproducibility of antigenic preparations that often rely on outdated methodologies, improperly defined as “purifications”. This adds to the use of different panels of sera, as well as to the lack of clinical characterization and appropriate classification/grouping of sera, used for validation [17–19].

Among HCF antigens, Antigen 5 (Ag5) and Antigen B (AgB) are the most abundant and immunogenic proteins, whose role in the life cycle of the cestode has been assessed only partially [20]. In a recent work [21], our research group reported a straightforward, robust and reproducible chromatography method that enables the preparation of a HCF fraction highly enriched in native Ag5. This highly enriched antigen demonstrated a strong reactivity, both in western blotting and ELISA formats, when tested on a limited panel of sera from CE patients, encouraging a more extensive examination of its diagnostic potential.

Here we present a large-scale study (327 cases and 253 controls) aimed to evaluate the diagnostic accuracy of two different Ag5 ELISA setups compared with that of a commercially available ELISA widely employed for routine diagnostic. We also investigated the association between readings of the Ag5-based ELISAs with selected clinical variables of the patients. When the three assays were compared for sensitivity and specificity, the Ag5 ELISA had significantly higher sensitivity. Moreover, the performances of both Ag5 ELISA setups were statistically associated with clinical variables known to influence serology results. These data support the use of this Ag5-based preparation in highly sensitive diagnostic tests and prompt further investigation on its use in the follow-up of CE patients.

## Methods

### Ethics statement

A written informed consent to use of leftover serum after routine serology for research was obtained from patients at the time of sample collection. The study was approved by the ethics committees of IRCCS San Matteo Hospital Foundation, Pavia, Italy, Prot N. 20150004877, for sera from CE patients, and of the local health authority of Sassari (ASL N. 1, Sassari), Prot N. 1123/L, for sera from healthy controls.

### Patients and samples

A prospective study was performed on sera from patients with hepatic and extra-hepatic CE, collected at the Department of Infectious Diseases of the IRCCS San Matteo Hospital Foundation, Pavia, Italy, where the WHO Collaborating Centre for Clinical Management of Cystic Echinococcosis is based. Sera from healthy subjects, collected at the Sassari Hospital Blood Donor Center, Sassari, Italy, were used as a control group. At the time of serum collection, all patients with abdominal cysts were diagnosed by US and CE cysts were classified according to the World Health Organization–Informal Working Group on Echinococcosis (WHO-IWGE) standardized US classification, by a clinician with long standing experience in the US diagnosis of CE, as part of the routine diagnostic procedures. This classification groups cysts in six stages based on a biological/dynamic approach: active (CE1 and CE2), transitional (CE3a and CE3b) and inactive (CE4 and CE5) cysts. However, CE3b are actually biologically active [8]. On the other hand, from a serological point of view, CE3 cysts of both stages often show comparable results, indicating that biological activity at the time of serum collection may not immediately influence serological responses in these stages [13]. Therefore, for the purpose of analysis, sera from patients with active (CE1, CE2 and CE3b) and transitional (CE3a) cysts were grouped together.

Patients having multiple cysts in different stages were assigned to the group of the most active cyst, independently from its hepatic or extra-hepatic localization.

### Sheep HCF

HCF crude samples were collected in two different Sardinian slaughterhouses (CE/IT2383M, Tula, Sassari and CE/IT2078M, Lula, Nuoro). Fluid was aspirated from liver and lung cysts found in infected sheep. The hydatid fluid was centrifuged at 1000 *g* at 4°C and the supernatant stored at -80°C.

### Antigen 5 preparation

Enriched Ag5 was obtained as described previously [21]. Briefly, after desalting and concentration, aliquots of sheep HCF were fractionated by Fast Protein Liquid Chromatography (FPLC) on a Superdex-200 column (10/300 GL, GE Healthcare, Uppsala, Sweden). The fractions of interest were pooled and their protein content was evaluated by tandem mass spectrometry on a Q-TOF hybrid mass spectrometer equipped with a nano lock Z-spray source and coupled on-line with a nanoAcquity chromatography system (Waters, Manchester, UK) to verify the quality of the preparation.

### Commercial ELISA test

Sera were tested in duplicate in the parasitology diagnostic laboratory of the IRCCS San Matteo Hospital Foundation, Pavia, Italy, by laboratory personnel with long standing experience in diagnostic parasitology, using a commercial ELISA test (RIDASCREEN *Echinococcus* IgG, R-Biopharm, Darmstadt, Germany), for the detection of *Echinococcus* specific total IgG, according to manufacturer’s instructions.

Tests were read at 450nm in a spectrophotometer, and a Sample Index (SI) was calculated and interpreted for each serum according to manufacturer’s instructions; ELISA was considered positive for SI >1.1, negative for SI <0.9, and border line for 0.9 ≤SI ≤1.1. However, for statistical evaluations, borderline results were classified together with negatives. Readers were blind to the results of the other tests.

### Ag 5 ELISA

Sera were tested with the Ag5 ELISAs based on our Ag5 enriched preparation [21], following two alternative setups, A and B, in the laboratory of Porto Conte Ricerche, Alghero, Italy. Briefly, for setup A, microplates (Nunc-Maxisorp Immunoplate, Waltham, Massachusetts, USA) were coated with 100 μL/well of a 100 ng/mL antigen solution in phosphate buffered saline (PBS). After blocking and washings, sera were added at 1:500 dilution in 2% bovine serum albumin in PBS-0.05% tween-20 (BSA in PBS-0.05%T) and incubated at 37°C for 1 hour. For setup B, microplates were coated with 100 μL/well of a 50 ng/mL antigen solution in PBS, and sera were added at 1:200 dilution. In both cases, secondary antibody (horseradish peroxidase conjugated anti-human IgG, Sigma-Aldrich, St. Louis, MO, USA) was diluted 1:100,000 in 2% BSA in PBS-0.05%T and incubated at 37°C for 1 hour. Finally, the substrate (3,3',5,5'-Tetramethylbenzidine Liquid Substrate, Supersensitive, Sigma) was added. The absorbance was read at 620 nm after 1 hour incubation using a Tecan Sunrise (Tecan Group, Ltd., Männedorf, Switzerland) microplate reader. All sera were tested in duplicate. In order to compare results obtained from different plates, a Sample Ratio (SR) was calculated according to the following formula:

$$SR=\frac{\mathrm{Sample mean}-\mathrm{Negative control mean}}{\mathrm{Positive control mean}-\mathrm{Negative control mean}}$$

where the negative control was a pool of fifteen healthy donors and the positive control was the Working Standard Anti-Echinococcus Serum, Human (NIBSC, Potters Bar, England).

Reproducibility among different Ag5 batches was evaluated for both setup A and setup B on three different, independent preparations. Ten sera (high, medium and low positive control samples) were tested in duplicate and results were evaluated by calculating the mean coefficient of variation (CV) among the three tests, for each setup.

All measurements were carried out in parallel by two experienced research fellows. Readers were blind to the results of the other tests.

### Statistical analysis

Data analysis was performed with MedCalc Statistical Software version 15.2.2 (MedCalc Software bvba, Ostend, Belgium; http://www.medcalc.org; 2015). A receiver-operator characteristic analysis (ROC) [22–23] was performed to determine a cut-off value for each Ag5 based test. The standard error and the area under the curve were calculated according to DeLong et al. [24]. Levels of sensitivity were plotted against levels of 100 minus specificity at each cut-off point on a ROC curve. Threshold values used were those associated with the highest Youden index J [25]. In order to calculate the best ELISA cut-off values, and to improve sensitivity on active-transitional cysts, that are generally seropositive with HCF-based tests (while patients with CE4 and CE5 cysts and post-surgical patients have most commonly negative or low results), ROC curves were built by using SR values from patients with CE1, CE2, CE3a and CE3b as positive group (171 sera) and healthy controls as negative group (253 sera). The area under the ROC curve (AUC) was used to define the antigen discriminatory power (between subjects with active-transitional cysts and subjects with inactive cysts or without the disease). A *p*-value <0.05 was considered statistically significant. McNemar test was performed, on all 580 sera, to compare the sensitivities of the two in-house Ag5 setups and the commercial assays. Differences in SR or SI values between groups were analyzed by Kruskal-Wallis test, for the three ELISAs, independently; when more than two groups were analyzed, after Kruskal-Wallis test, pairwise multiple comparisons were evaluated by Conover test with Bonferroni correction [26–27]. Inter-rater agreement test was used to evaluate the agreement between the gold standard (US) and the in-house ELISAs, and the results were expressed by Kappa (K) statistic, with 95% confidence interval [28]. Moreover, three statistical analyses were performed on the results of sera from CE patients to assess the effect of the cyst stage, the number of cysts and the previous treatment with albendazole (ABZ), potentially affecting the assay performance of Ag5 ELISAs [13,18]. While cyst localization is an important variable and its influence on ELISA results is potentially interesting, it was not evaluated given the low number of patients with extra hepatic cysts. Chi squared test was applied to compare the sensitivity of the two Ag5 ELISA setups within subgroups of patients, classified according to their clinical variables. Then, the same variables were further evaluated by a bivariate logistic regression, to consider their influence on test performances and calculate an odd ratio (OR) for each pair of variables. Finally, all the examined clinical variables were concurrently analyzed by a multiple regression, to evaluate their relationship on the diagnostic result. A *p*-value <0.05 was considered statistically significant.

## Results

### Sera from CE patients

A total of 580 blood sera were collected from June 2008 to June 2012, including 283 from patients with CE cysts, 44 during follow-up after surgery, and 253 from healthy subjects. As summarized in Table 1, 295 sera were collected from patients with hepatic cysts (90.2%), one serum from a pulmonary case (0.3%), and the remaining 31 sera (9.5%) were from patients with other localizations (including peritoneum, kidney, and leg); single cysts were found in 146 patients (44.6%), whilst 2 or more cysts were found in the other 137 subjects.

**Table 1:** Patients grouping according to clinical characteristics and localization of the most active cyst.

Group	Number of patients	Hepatic cysts	Extra hepatic cysts
CE1	15	10 (66.7%)	5 (33.3%)
CE2	9	9 (100%)	0
CE3a	40	35 (87.5%)	5 (12.5%)
CE3b	107	98 (91.6%)	9 (8.4%)
CE4	76	74 (97.4%)	2 (2.6%)
CE5	36	33 (91.7%)	3 (8.3%)
Post-surgery	44	36 (81.8%)	8 (18.2%)
Total	327	90.2%	9.8%

### Serology assays by Ag5 and commercial ELISA

**Fig 1:**
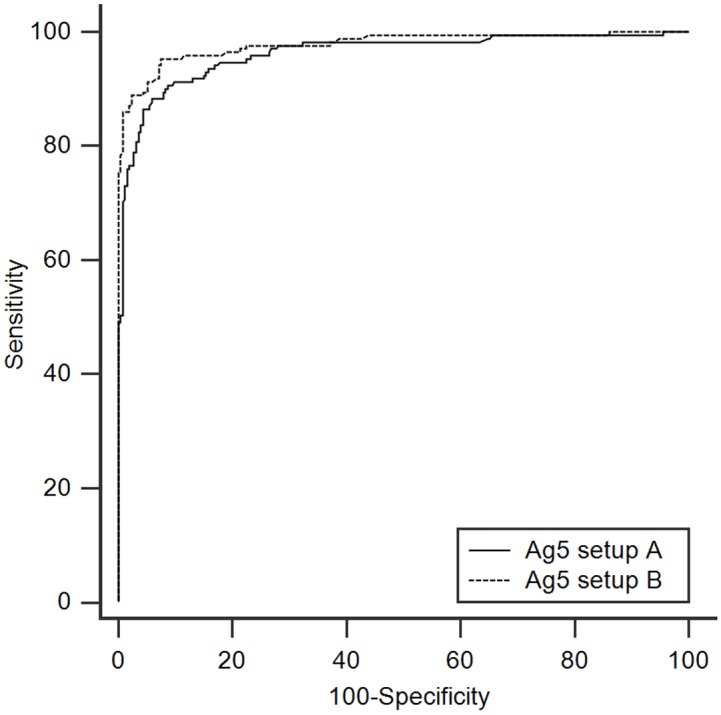
**ROC curves overlay of the Ag5 ELISA setup A (solid line) and B (dashed line).** Curves are generated by plotting Sensitivity versus 100-Specificity.

The reproducibility among Ag5 lots was assessed in both setups, demonstrating the reliable performances of our Ag5 preparation. Specifically, setup A had a CV of 9.7%, and setup B had a CV of 11.3%. All sera were analyzed by the commercial assay RIDASCREEN and by the two experimental Ag5 ELISA setups, from November 2011 to September 2012. Then, Ag5 ELISAs were evaluated by ROC curves (Fig 1 and Table 2) to define optimal cut-off values for data analysis. The areas under the ROC curve (AUC) were 0.962 and 0.978 for setup A and setup B, respectively. Serological results and their statistical significance are summarized in Tables 3 and S1. At the best cut off value (0.261 and 0.120 for setup A and setup B, respectively), the two Ag5 ELISA setups showed different sensitivity. In particular, Ag5 ELISA setup B revealed an overall sensitivity higher than both Ag5 setup A and RIDASCREEN test, whilst Ag5 setup A showed similar sensitivity to the commercial kit. More in detail, Ag5 setup B results displayed statistically significant differences when CE3b, CE4 and post surgery patients were examined. These differences persisted when grouping patients as active-transitional and inactive. Concerning the control sera, a higher number of donors tested positive in Ag5 setup B (7.5%), followed by Ag5 setup A (5.9%) and RIDASCREEN (1.6%). Hence, the Ag5 ELISA, especially in setup B, revealed a higher sensitivity and a lower specificity than the commercial RIDASCREEN test.

**Table 2:** Statistical parameters of the Ag5 ELISA setups at the best cut-off values.

Ag5 setup	% Sensitivity	% Specificity	AUC (±SE)	AUC CI (95%)	Youden index	(+LR)	(-LR)
A	88.3	94.1	0.962 (±0.0095)	0.940–0.978	0.824	14.89	0.12
B	95.3	92.5	0.978 (±0.0071)	0.959–0.990	0.878	12.69	0.051

AUC: Area Under the Curve. SE: Standard Error. CI: Confidence Interval. +LR: Positive Likelihood Ratio.–LR: Negative Likelihood Ratio.

**Table 3:** ELISA serological results.

Group		N. of patients	Positive by Ag5 setup A	Positive by Ag5 setup B	Positive by RIDASCREEN
**Active-Transitional**	**Total**	**171**	**151 (88.3%; B)**	**163 (95.3%; A, R)**	**150 (87.7%; B)**
	CE1	15	12 (80.0%)	14 (93.3%)	10 (66.7%)
	CE2	9	9 (100%)	9 (100%)	9 (100%)
	CE3a	40	38 (95.0%)	40 (100%)	40 (100%)
	CE3b	107	92 (86.0%; B)	100 (93.4%; A, R)	91 (85%; B)
**Inactive**	**Total**	**112**	**71 (63.4%; B)**	**84 (75.0%; A, R)**	**72 (64.3%; B)**
	CE4	76	54 (71.0%; B)	63 (82.9%; A, R)	56 (73.7%; B)
	CE5	36	17 (47.2%)	21 (58.3%)	16 (41.7%)
**Post-surgery**	**Total**	**44**	**18 (40.9%; B)**	**32 (72.7%; A, R)**	**19 (43.2%; B)**
**Healthy controls**	**Total**	**253**	**15 (5.9%; R)**	**19 (7.5%; R)**	**4 (1.6%; A, B)**

A, B, R indicate statistically significant differences (p-value <0.05) between tests, according to McNemar test (A: different from Ag5 setup A; B: different from Ag5 setup B; R: different from RIDASCREEN).

**Fig 2:**
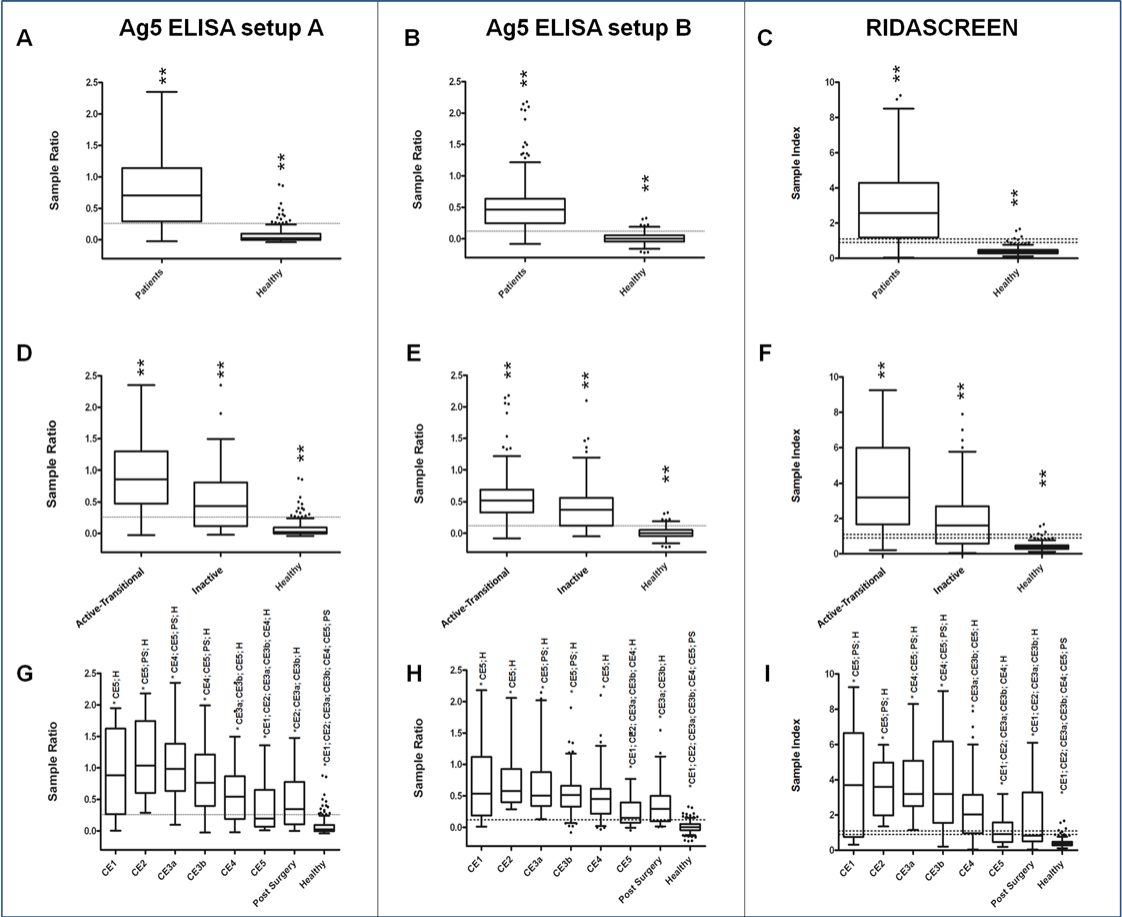
**Box-and-whiskers plots of ELISA results for Ag5 setup A, Ag5 setup B, and RIDASCREEN, (left, middle, and right panels, respectively).** Boxes indicate values falling within the 25^th^ and 75^th^ percentiles (InterQuartile Range, IQR). Central lines represent the median values. Whiskers indicate values falling within the ±1.5*IQR. Single dots depict values falling outside the whiskers. Dashed lines indicate the best cut-off values, except for the right panels, where lower and upper limits for borderline values are reported. ** = statistically different groups after Kruskal-Wallis test, or after Conover test, with Bonferroni correction, for multiple comparisons. According to this adjustment, to achieve statistical significance three different *p*-value have been considered: *p* <0.05 for panels A, B, and C; *p* <0.017 for panels D, E, and F; *p* <0.0018 for panels G, H, and I. Groups significantly different in panels G, H, and I are indicated as (* group). P: patients; PS: post surgery; H: healthy donors.

The comparison among the Ag5 ELISAs described in this work and the commercial ELISA is also plotted in Fig 2. Although it should be noted that the ELISA values are not directly comparable due to the difference in OD normalization, Kruskal-Wallis test on SR or SI values confirmed that all the three ELISAs were able to discriminate between patients and healthy controls (Fig 2A, 2B and 2C); statistically different results were also obtained with the three ELISAs, when patients were grouped taking into account the active-transitional versus the inactive stages of CE (Fig 2D, 2E and 2F). Finally, none of the three methods was able to completely discriminate among any of the CE groups and post surgery follow-up patients (Fig 2G, 2H and 2I); however, pairwise comparisons of the subgroups highlighted some differences. Both CE1 and CE2 cysts were different from CE5 in the three assays; CE3a and CE3b were always comparable, but behaved differently in the three tests. The inactive cysts showed important differences in all the tests: CE4 revealed SR or SI values statistically different from CE3a, CE3b and CE5 in Ag5 setup A and RIDASCREEN ELISAs, whilst it differed only from CE5 in Ag5 setup B. CE5, on the contrary, performed differently from all the other groups (except for post surgery patients) in all the ELISAs. Finally, post surgery patients showed dissimilar behavior in all the tests, although with a higher divergence from active-transitional groups for RIDASCREEN results.

Further, the Ag5 setup A and the commercial kit, provided a wider range of antibody levels, whilst for Ag5 setup B results were concentrated in a narrower range. The agreement between the gold standard method (US) and our Ag5 ELISAs was evaluated by inter-rater agreement (Kappa) test. When considering patients with CE1, CE2, CE3a and CE3b, kappa (K) value was 0.828, with a standard error of 0.0279 (setup A), or 0.869, with a standard error of 0.0243 (setup B), confirming the excellent agreement between the imaging diagnosis and the ELISA results. When CE4 and CE5 patients were also included in the test, this agreement was poorer, with a K value of 0.718, with a standard error of 0.0295 (setup A), or 0.795, with a standard error of 0.0262 (setup B). This is not surprising, since these patients, due to the inactivity of the cysts, are often negative to ELISA.

In addition to the cyst stage, other major clinical variables such as the number of cysts and the previous ABZ treatment were taken into account using Chi squared test to assess the ability of the Ag5 ELISAs to discriminate among CE patients with different clinical traits. Results are summarized in Table 4. For both Ag5 ELISA setups, this test showed a statistical significance (*p*-value <0.05) for the stratification of patients in terms of single vs multiple cysts, active-transitional vs inactive cysts and for current or past ABZ treatment vs no ABZ. More in detail, pharmacologically treated patients gave positive results to both Ag5 setups more frequently than untreated patients; further, patients in the active or transitional stage had higher positivity rates than patients in the inactive stages; finally, patients with more than one cyst were positive to Ag5 ELISAs more frequently than patients with one cyst.

**Table 4:** Influence of clinical variables on Ag5 ELISA outcomes.

Variable		Chi Squared test				Logistic Regression		Multiple Regression	
		Ag5 setup A		Ag5 setup B		Ag5 setup A	Ag5 setup B	Ag5 setup A	Ag5 setup B
		Positive	*p*-value	Positive	*p*-value	*p*-value (OR)	*p*-value (OR)	*p*-value	*p*-value
ABZ treatment	Yes (n = 205)	186 (90.7%)	**<0.0001**	194 (94.6%)	**<0.0001**	**<0.0001 (9.82)**	**<0.0001(6.22)**	**<0.0001**	**<0.0001**
	No (n = 78)	36 (46.1%)		53 (67.9%)					
Cyst stage	CE1-CE2-CE3a-CE3b (n = 171)	151 (88.3%)	**<0.0001**	163 (95.3%)	**<0.0001**	**0.0002 (3.68)**	**0.0002 (5.34)**	**0.0001**	**<0.0001**
	CE4-CE5 (n = 112)	71 (63.4%)		84 (75.0%)					
Number of cysts	Single (n = 146)	103 (70.5%)	**0.0014**	117 (80.1%)	**0.0004**	0.055 (1.99)	**0.0101 (3.39)**	0.0518	**0.0084**
	Multiple (n = 137)	119 (86.9%)		130 (94.9%)					

Bold characters indicate statistically significant differences (*p*-value <0.05). OR: Odds Ratios

All the above-mentioned clinical variables were evaluated by a bivariate logistic regression. The statistical significance persisted, with fairly high values of odds ratios, when grouping patients according to active-transitional vs inactive cysts and for ABZ treatment vs no ABZ, but it became only borderline significant for Ag5 setup A, when single vs multiple cysts were considered. The interdependence of test results from the pharmacological treatment and the activity of the cysts was confirmed for both Ag5 ELISA setups by multiple regression; the effect of the number of cysts, instead, was only borderline significant for setup A (*p*-value = 0.055), remaining significant for setup B.

## Discussion

CE is a public health and economic issue, concerning both humans and farm animals, and requires an early and unambiguous diagnosis. Imaging techniques remain the most reliable method for an accurate diagnosis. Serological tests are required for diagnosis confirmation in doubtful cases, but their current sensitivity and specificity are unsatisfactory, while their value in the monitoring of patients during follow-up is very limited. The development of robust and stage-specific serological tests is therefore still needed. Currently, commercially available serological kits are based on western blotting, hemagglutination, and ELISA, and mostly use HCF as target antigen, a complex mixture of host and parasite electrolytes, proteins, nitrogenous waste products, carbohydrates and lipids. Its composition is known to vary, often significantly, from cyst to cyst [21,29–30]. As a consequence, sensitivity and specificity are very heterogeneous across tests. Technological improvements have provided increasingly reliable antigens and tools [14,16,18,31–35], but their performances are still suboptimal and their production is often expensive or patented, limiting their use in the most affected regions, which are often developing countries that cannot afford the appropriate facilities [17]. Ag5 and AgB are reported to be the most abundant and immunogenic proteins in the cyst fluid [20]. After an initial growing interest in the use of Ag5 for diagnostics, the focus has moved mostly towards AgB, and its subunits. However, it is reported that a proportion of CE patients with active cysts do not develop a detectable humoral response against AgB [21,36]. Ag5 cross-reactivity issues, as well as low sensitivity and specificity, have been discussed in many papers [16,37–38], and they are probably the main reason for the decline in Ag5 use for CE immunodiagnosis. Part of the cross-reactivity was associated with the presence of phosphorylcholine bound to the Ag5 38 kDa subunit [39–40]. On the other hand, Ag5 protein shares 96.7% and 85.5% identity with the homologous sequences of *Taenia solium* and *Echinococcus multilocularis*, respectively, and it is inevitable that the same epitopes are present on these proteins. However, interestingly, Ahn and coworkers [30] showed that Ag5 seems to be immunoreactive in every stage of the pathology, as opposed to AgB, whose proteoforms revealed a reduced antibody capturing activity in CE1, CE4 and CE5 stages. Further studies using sera from patients with other relevant parasitoses are needed to assess the behavior of our Ag5-based ELISAs and to evaluate the value of Ag5-based assays for patient follow-up. Concerning the low sensitivity and specificity reported in previous works, it must be underlined that all the experimental studies concerning Ag5 date back to decades ago, when the analytical techniques themselves suffered from low sensitivity. Therefore, it is likely that the low diagnostic performance reported so far for tests based on Ag5 could be explained with the high heterogeneity of the antigen preparations used at that time. Our results show that Ag5 is a sensitive antigen and further studies using sera from patients with non-CE solid lesions are warranted to evaluate and optimize the cut-off value of the ELISAs when higher sensitivity is needed in the differential diagnosis of inactive cysts.

In a previous work [21], a chromatographic method for the reproducible preparation of native Ag5 from different HCF sources was described, enabling the production of a protein fraction highly enriched in Ag5, as verified by mass spectrometry. Preliminary ELISA experiments on a limited panel of CE sera revealed the high reactivity of this antigen. Therefore, we were prompted to evaluate the diagnostic performance of this antigen preparation on a substantial number of CE patients and healthy control sera. ROC curves generated by both Ag5 ELISA setups using CE1, CE2, CE3a and CE3b sera demonstrated its good sensitivity and specificity. When comparing the diagnostic accuracy of these Ag5-based ELISAs with a commercial kit routinely used in clinical laboratories, the excellent performances for Ag5 setup B outperformed those of the commercial test. Nevertheless, specificity was higher for the commercial kit.

When we evaluated the effect of clinical variables on Ag5 ELISA results, we found that patients with more than one cyst, and/or in the active or transitional stage, and/or who received drug therapy, were positive to Ag5 test more frequently than the other patients. The bivariate logistic regression and the multiple regression both highlighted an effect due to the pharmacological treatment and to the cyst activity, while the number of cysts maintained a statistical significance only when setup B was used, confirming the importance of these variables as reported in other previous works [13,18,35].

The biological mechanism at the basis of the influence of ABZ treatment on serology results was attributed to high levels of ABZ in cyst fluid causing the germinal laminated membranes to become more permeable, inducing a leakage of their antigenic contents in the blood stream. In turn, the leakage of parasite antigens triggers and sustains increased concentration of circulating antibodies[41–42]. Concerning the effect of the number of cysts and the stage of the disease, no experimental study has demonstrated the biological mechanisms underlying these serology patterns, however it is likely that the loss of cyst wall integrity during the evolution of the cyst and the presence of a large antigenic mass when multiple cysts are present may explain the observed serology behavior.

In conclusion, the described serological assay, combining robustness, sensitivity, and easiness of execution, with the low cost, high reproducibility and rapidity of the Ag5 preparation method, makes this antigen a promising candidate for the serodiagnosis of CE especially in the setup B. Moreover, to our knowledge, this is the first report on the influence of the pharmacological treatment, the cyst stage, and the number of cysts on the results of an Ag5-based ELISA test.

## Supporting Information

S1 Table Statistical significance of inter-test sensitivity comparison. (DOCX)
