# Supplementary material for: Diagnostic Accuracy of Antigen 5-Based ELISAs for Human Cystic Echinococcosis
Source: PLoS Negl Trop Dis. 2016 Mar 29;10(3):e0004585. doi: 10.1371/journal.pntd.0004585 (PMC4811537; doi:10.1371/journal.pntd.0004585)
Supplement: S1 Table — (DOCX) [file pntd.0004585.s001.docx]

**Table S1**. Statistical significance of inter-test sensitivity comparison.

| Group | | N° of patients | Ag5 setup A  vs  Ag5 setup B | Ag5 setup A  vs  RIDASCREEN | Ag5 setup B  vs  RIDASCREEN |
| --- | --- | --- | --- | --- | --- |
| Active-Transitional | CE1 | 15 | *p* = 0.5000 | *p* = 0.5000 | *p* = 0.1250 |
|  | CE2 | 9 | ND | ND | ND |
|  | CE3a | 40 | *p* = 0.5000 | *p* = 0.5000 | ND |
|  | CE3b | 107 | ***p* = 0.0386** | *p* = 1.0000 | ***p* = 0.0352** |
|  | CE1-CE2-CE3a-CE3b | 171 | ***p* = 0.0042** | *p* = 1.0000 | ***p* = 0.0044** |
| Inactive | CE4 | 76 | ***p* = 0.0039** | *p* = 0.7744 | ***p* = 0.0391** |
|  | CE5 | 36 | *p* = 0.2891 | *p* = 1.0000 | *p* = 0.1797 |
|  | CE4-CE5 | 112 | ***p* = 0.0023** | *p* = 1.0000 | ***p* = 0.0075** |
| Post-surgery | | 44 | ***p* = 0.0313** | *p* = 0.1094 | ***p* = 0.0005** |
| Healthy control | | 253 | *p* = 0.4862 | ***p* = 0.0042** | ***p* = 0.0002** |

Bold characters indicate statistically significant differences (*p*-value <0.05) according to McNemar test. ND: No difference.
